# Hydrotropic Hydrogels Prepared from Polyglycerol Dendrimers: Enhanced Solubilization and Release of Paclitaxel

**DOI:** 10.3390/gels8100614

**Published:** 2022-09-26

**Authors:** Tooru Ooya, Jaehwi Lee

**Affiliations:** 1 Department of Chemical Science and Engineering, Graduate School of Engineering, Kobe University, 1-1 Rokkodai-cho, Nada-ku, Kobe 657-8501, Japan; 2 Center for Advanced Medical Engineering Research & Development (CAMED), Kobe University, 1-5-1 Minatojima-Minamimachi, Kobe 657-0047, Japan; 3 College of Pharmacy, Chung-Ang University, 84 Heukseok-ro, Dongjak-gu, Seoul 06974, Korea

**Keywords:** polyglycerols, dendrimers, hydrogels, paclitaxel, release, solubilization, FTIR imaging, hydrotrope

## Abstract

Polyglycerol dendrimers (PGD) exhibit unique properties such as drug delivery, drug solubilization, bioimaging, and diagnostics. In this study, PGD hydrogels were prepared and evaluated as devices for controlled drug release with good solubilization properties. The PGD hydrogels were prepared by crosslinking using ethylene glycol diglycidylether (EGDGE). The concentrations of EGDGE and PGDs were varied. The hydrogels were swellable in ethanol for loading paclitaxel (PTX). The amount of PTX in the hydrogels increased with the swelling ratio, which is proportional to EGDGE/OH ratio, meaning that heterogeneous crosslinking of PGD made high dense region of PGD molecules in the matrix. The hydrogels remained transparent after loading PTX and standing in water for one day, indicating that PTX was dispersed in the hydrogels without any crystallization in water. The results of FTIR imaging of the PTX-loaded PGD hydrogels revealed good dispersion of PTX in the hydrogel matrix. Sixty percent of the loaded PTX was released in a sink condition within 90 min, suggesting that the solubilized PTX would be useful for controlled release without any precipitation. Polyglycerol dendrimer hydrogels are expected to be applicable for rapid release of poorly water-soluble drugs, e.g., for oral administration.

## 1. Introduction

**Figure 1:**
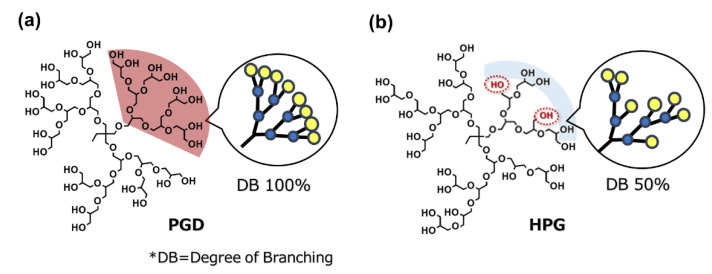
Representative structure of (**a**) PGD and (**b**) HPG; OH groups in the dotted circles constructs liner parts, resulting in decreasing DB.

Dendritic glycerol is a glycerol molecule with a branched chemical structure similar to those of polyglycerol dendrimer (PGD) (Figure 1a) and hyperbranched polyglycerol (HPG) (Figure 1b). Dendritic glycerol consists of a polyether structure, similar to the highly biocompatible polyethylene glycol (PEG), as the backbone with a branched structure, and has many hydroxyl groups at the ends [1,2,3]. In addition to its high water solubility and biocompatibility, it has advantages such as higher thermal stability compared to PEG [4]. One reason for using PGDs in bio-applications is that the single molecular weight and monodisperse nature of PGDs with perfect degree of branching (DB) make them suitable for targeted modification of molecular ends. In addition, the ability to precisely control the molecular weight allows for a detailed examination of the effects of molecular weight, molecular size, and number of end groups on the system. Hyperbranched polyglycerol is easy to synthesize and can generate high-molecular-weight polymers in a single step, which is difficult with the stepwise organic synthesis that is required for PGD. To date, PGDs and HPGs have been used as nanocapsules for drug delivery [5,6], dispersions of metal ions using host–guest interactions [7], protein adsorption inhibitory surfaces [8,9,10,11,12], hydrogels [13,14,15,16,17], human serum albumin substitutes [18], organ preservation solutions [19], and solvents for poorly water soluble drugs as hydrotropes [20,21,22,23,24,25,26].

Hydrogels are composed of a three-dimensional polymer network formed by cross-linked hydrophilic polymer chains. They are insoluble in water and can hold a large amount of internal water [27,28]. The preparation of hydrogels using HPG has been reported by Oudshoorn et al. The gelation is carried out using potassium persulfate as a radical initiator [13]. Other crosslinking methods include enzymatic catalysis [14] and biomimetic mineralization [15]. The expected advantages of HPG hydrogels are that the low viscosity of HPG in water enables the preparation of hydrogels with high polymer concentrations and controlled mechanical properties [29]. Hyperbranched polyglycerol hydrogels have reportedly been used as bioinks for microfabrication [30], scaffolds for living cells [14,31,32], and drug delivery systems for poorly-water-soluble drugs and proteins [33]. However, PGD-crosslinked hydrogels have not been reported so far.

We have shown that PGDs are a hydrotrope for paclitaxel (PTX; 5β,20-Epoxy-1,2α,4,7β,13α-hexahydroxytax-11-en-9-one4,10-diacetate2-benzoate13-ester with (2*R*,3*S*)-*N*-benzoyl-3-phenyllisoserine, molecular formula C_47_H_51_NO_14,_ corresponding to molecular weight of 853.91 Da). Paclitaxel is a well-known anti-tumor agent with poor water solubility (0.6 ± 0.08 μg/mL [34]), so that it is clinically formulated in a mixture composed of 1:1 blend of Cremophor EL (polyethoxylated castor oil) and ethanol [35]. Paclitaxel promotes the assembly of microtubles, resulting in cancer cells death via protecting from the disassembly of microtubules induced by cold or calcium treatment [36]. Polyethylene glycol 400 (PEG400), which is known as a co-solvent for dissolving PTX at high concentration, requires about 50 wt% to dissolve 0.1 mg/mL of PTX, while about 10 wt% is sufficient for PGD [20]. Furthermore, precipitation occurs upon dilution, suggesting that PGD does not incorporate PTX but functions as a “hydrotropic” molecule that dissolves PTX by interacting with the surrounding PTX molecules. In order to apply this dissolution property, the dissolved PTX release must be controlled. In the present study, PGD-crosslinked hydrogels were prepared by using ethylene glycol diglycidylether (EGDGE). If the local concentration of PGDs can be increased by chemical crosslinking, the solubility of PTX is expected to increase. Crosslinking conditions such as the solvent and concentration were varied, and the obtained hydrogels were evaluated in terms of swelling, PTX loading, dissolution state of PTX, and release of PTX.

## 2. Results and Discussion

**Figure 2:**
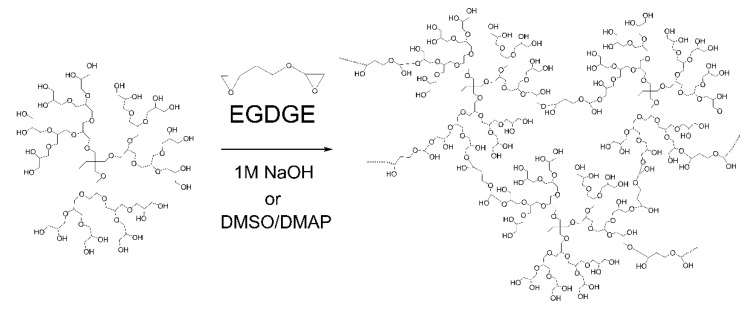
Synthetic scheme of PGD-G3 hydrogels using EGDGE as crosslinking agent (G3-EG hydrogels).

**Figure 3:**
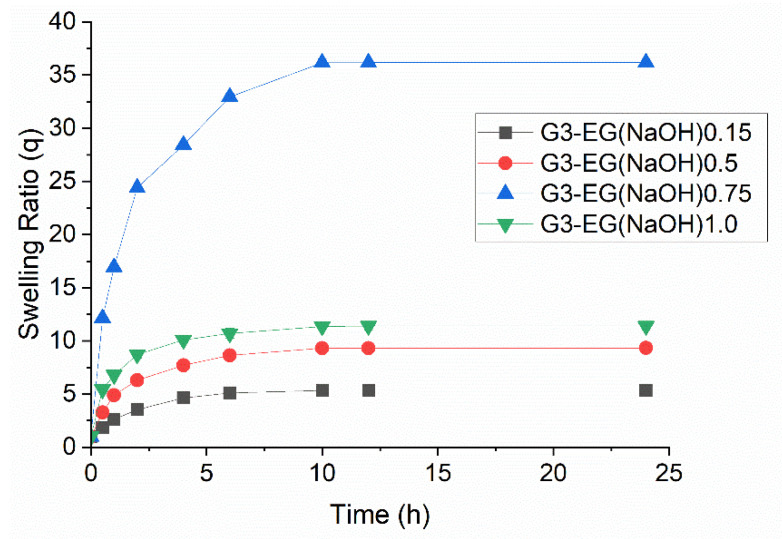
Swelling behavior of G3-EG hydrogels in water at 25 °C: black: G3-EG(NaOH)0.15, red: G3-RG(NaOH)0.15, blue: G3-EG(NaOH)0.75, green: G3-EG(NaOH)1.0. Detailed preparation conditions of each hydrogel are summarized in Table 1 (mean ± S.E.M., n = 3).

Polyglycerol dendrimer of generation 3 (PGD-G3)was crosslinked by reaction with EGDGE (Figure 2). It is well-known that hydroxyl groups in water-soluble polysaccharides can be modified by glycidyl ethers in NaOH aqueous solution [37] and in DMSO in the presence of appropriate catalysts such as DMAP [38]. Since PGD-G3 has many hydroxyl groups, these methods are applicable to the preparation of crosslinked PGD-G3 hydrogels. As shown in Table 1, the concentrations of EGDGE and PGD-G3 were varied. Since the solubility of EGDGE in 1 M NaOH solution is limited, additional 1 M NaOH was added to the reaction mixture when the ratio of EGDGE and hydroxyl groups of PGD-G3 was increased. This results in decreasing the final concentration of PGD-G3. The swelling ratio in water increased with increasing concentration of EGDGE and decreasing concentration of PGD-G3. All the hydrogels prepared in 1 M NaOH reached their equilibrium swelling at around 10 h (Figure 3). When both DMSO and DMAP were used for gel preparation, the concentrations of EGDGE and PGD-G3 both increased, and the swelling ratio for G3-EG(DMSO)0.75 was the lowest among all samples. All the hydrogels were stiff, except for G3-EG(NaOH)0.75, which could not maintain a disc shape. Taking the largest swelling ratio of G3-EG(NaOH)0.75 into account, the crosslinking condition for G3-EG(NaOH)0.75 induced partially intramolecular crosslinking of PGD-G3 molecules. The hydrogels were also swellable in ethanol, which allows loading of PTX.

In order to compare the influence of the generation of PGD, PGD-G4 hydrogels were prepared in a similar manner in NaOH (Table 2). The swelling ratio for each hydrogel was slightly smaller than that for each G3-EG hydrogel, even with similar concentrations of EGDGE and PGDs. This may be due to the denser network of hydroxyl groups that can act as a more hyperbranched structure on the nanoscale than those in PGD-G3. However, the tendency of swelling for PGD-G3 and PGD-G4 was the same.

**Figure 4:**
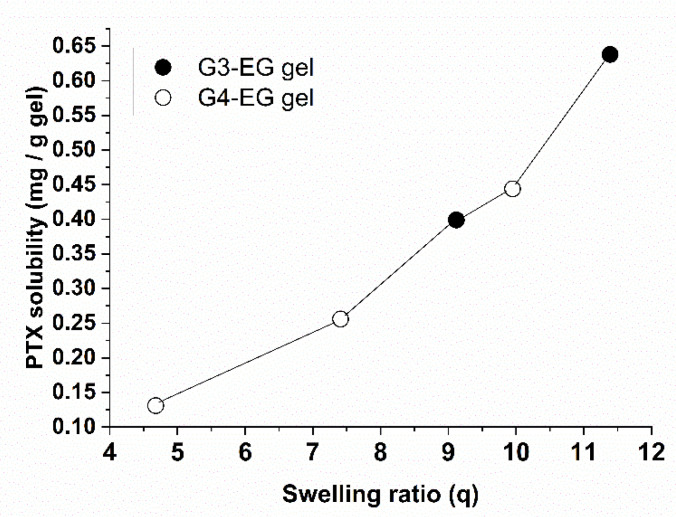
PTX solubility, determined by HPLC, per 1 g dried hydrogels (G3-EG(NaOH)0.5, G3-EG(NaOH)1.0, G4-EG(NaOH)0.15, G4-EG(NaOH)0.5, and G4-EG(NaOH)1.0) as a function of swelling ratio in water.

**Figure 5:**
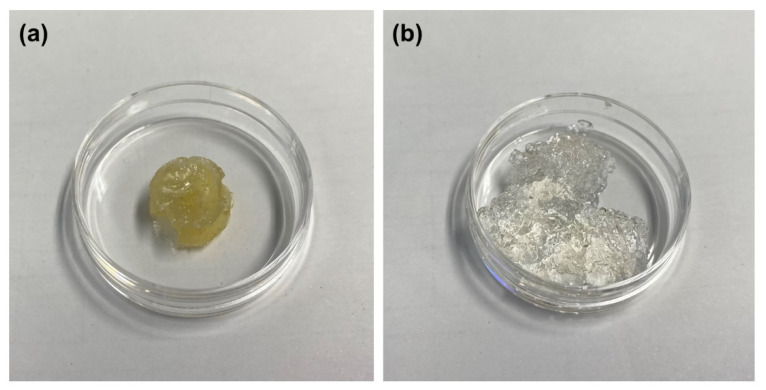
Photographs of (**a**) PTX-loaded dried G4-EG(NaOH)1.0 hydrogel and (**b**) swollen PTX-loaded G4-EG(NaOH)1.0 hydrogel. A small amount of water was added to G4-EG(NaOH)1.0 hydrogel after loading PTX, and the hydrogel was left to stand for one day.

Figure 4 shows the PTX amount loaded into hydrogels as a function of the swelling ratio in water. The PTX amount is proportional to the swelling ratio (except for G3-EG(NaOH)0.75 and G4-EG(NaOH)0.75 in Table 1 and Table 2), indicating that the dissolved PTX was entrapped in the spaces between the crosslinks in the swollen hydrogel. In other words, heterogeneous crosslinking of PGD made a high-density region of PGD molecules in the matrix, and this region is likely to act as a hydrotrope. To confirm the PTX solubility, a small amount of water was added to the G4-EG(NaOH)1.0 hydrogel after loading PTX to reach maximum swelling, and the gel was left to stand for one day. The hydrogel remained transparent although its shape collapsed (Figure 5), indicating that PTX was dispersed in the hydrogels without any crystallization in water.

**Figure 6:**
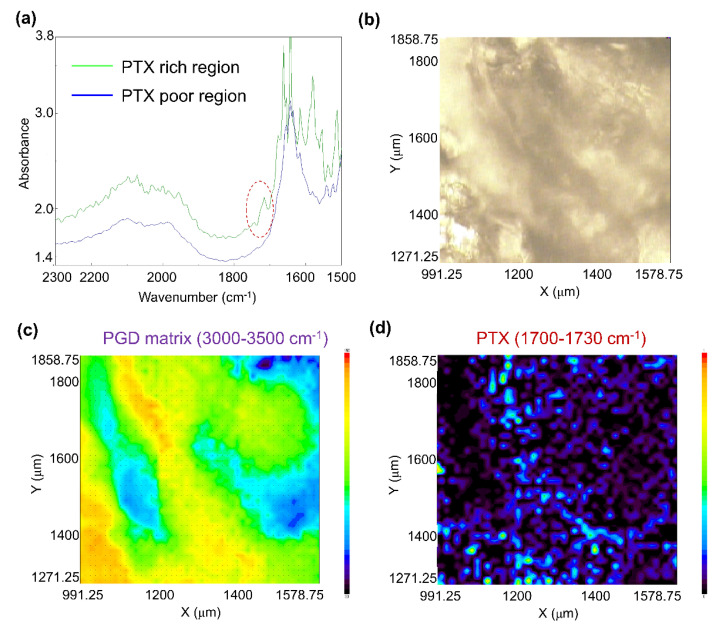
(**a**) Typical FTIR spectra of PTX-loaded dried G4-EG(NaOH)1.0 hydrogel (green line: PTX-rich region; blue line: PTX-poor region), (**b**) micrograph of PTX-loaded dried G4-EG(NaOH)1.0 hydrogel, (**c**) FTIR image of PTX-loaded dried G4-EG(NaOH)1.0 hydrogel using absorbance around 3000–3500 cm^−1^ (hydroxyl groups of PGD matrix), (**d**) FTIR image of PTX-loaded dried G4-EG(NaOH)1.0 hydrogel using absorbance around 1700–1730 cm^−1^ (carbonyl groups of PTX). Measurement area: 600 × 600 μm.

In order to check the degree of PTX dispersion, FTIR imaging was performed using the PTX-loaded dried G4-EG(NaOH)1.0 hydrogel. Typical FTIR spectra of PTX-rich and PTX-poor regions are shown in Figure 6a. Bands due to hydroxyl groups were observed from 3000 to 3500 cm^−1^ in both cases with the same absorbance (data not shown), which is consistent with a previous report on polyglycerol-based hydrogels [39]. However, the absorbance at 1700 to 1730 cm^−1^ increased in the PTX-rich region (see red circle in Figure 6a), suggesting the presence of carbonyl groups of PTX in the PTX-rich region. Based on these results, the absorbance values around 3000–3500 cm^−1^ and 1700–1730 cm^−1^ were adopted for 2D imaging of the PGD matrix and PTX distribution for 0.1 mm-thick gels (Figure 6b). As shown in Figure 6c, hydroxyl groups of PGD were not homogeneously distributed in the measured area, suggesting heterogeneous crosslinking by EGDGE. Interestingly, carbonyl groups of PTX were likely to be located in the PGD-rich region (Figure 6d). These results indicated that PTX was dispersed in the gel matrix, and it was assumed that PGD and PTX molecules interacted in the hydrogel and remained in a dissolved state.

**Figure 7:**
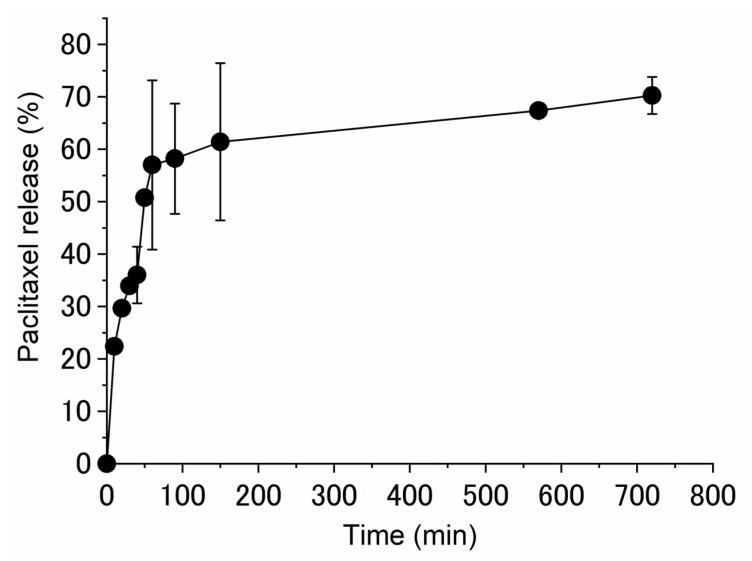
Cumulative release profiles for PTX loaded in G4-EG(NaOH)0.75 hydrogel. *N,N*-Diethylnicotinamide (1.5 M in PBS, pH 7.4) solution was used as the release medium to maintain an infinite sink condition. Samples were taken at predetermined time intervals and assayed for PTX by isocratic reverse-phase HPLC (mean ± S.E.M., n = 3).

Finally, PTX release from the PTX-loaded G4-EG(NaOH)1.0 hydrogel was evaluated in vitro. Approximately 60% of PTX was released in 90–150 min (Figure 7). Since PTX normally takes 1–2 days to be released when distributed and retained in the hydrophobic domain of micelles such as PEG-PLA block copolymers [40], these results suggest that the rapid release was governed by hydrotropic dissolution based on intermolecular interactions between PTX and the crosslinked PGD molecules. Under the release experimental conditions, PTX precipitation was not observed, and the hydrogel remained transparent even after 700 min. From this result, it is suggested that the remained PTX in the hydrogel (approximately 40%) was still solubilized in the hydrogel and entrapped in the G4-crosslinked matrix. The calculated amount of the released PTX at 150 min was 5.5 μg/mL, the concentration of which is reported to decrease cell viability to less than 40% using PTX-loaded nanoparticles against MCF-7 cells [41]. From these results and reports, the release of PTX can be achieved at the therapeutic level in vitro. We think that the PTX-loaded G4-EG or G3-EG hydrogels can be fabricated to nanogels by further chemical modification of OH-groups in combination with “click” chemistry [42] or the miniemulsion technique [43]. Such nanogels could be applicable for effective oral chemotherapy [41].

## 3. Conclusions

Polyglycerol dendrimers crosslinked by reacting with EGDGE exhibited an increased local concentration in the gel matrix, and PTX was successfully loaded into the hydrogels. The amount of loaded PTX was proportional to the swelling ratio of the hydrogels, regardless of the generation of PGD (G3 and G4). Fourier-transform infrared spectroscopic imaging of the PTX-loaded PGD hydrogels proved that PTX was retained and distributed in the hydrogel matrix after loading based on hydrotropic solubilization. Sixty percent of PTX was released in a few hours without recrystallization in the sink state, which is expected to be applicable for rapid release of PTX, e.g., for oral administration.

## 4. Materials and Methods

### 4.1. Materials

1,1,1-Tris(hydroxymethyl)propane (TMP) and dimethylaminopyridine (DMAP) were purchased from FUJIFILM Wako Pure Chemical Corporation (Osaka, Japan). Allyl chloride, *N*-methylmorpholine *N*-oxide (NMO), 50 wt.% sodium hydroxide solution, and paclitaxel (PTX) were purchased from Sigma-Aldrich (Tokyo, Japan). Tetrabutylammonium bromide, OsO_4_ (4% in water), and ethylene glycol diglycidylether (EGDGE) were purchased from Tokyo Chemical Industry Co., Ltd. (Tokyo, Japan). Generation 3 and 4 PGDs (PGD-G3: M_n_ = 1689, m/z 1712 [M + Na]^+^, calculated by MALDI-TOF-MS spectrometry (Voyager 2000, AB SCIEX); PGD-G4: M_n_ = 3508, m/z 3491 [M-H_2_O]) were prepared as described in previous papers [25,44].

### 4.2. Preparation of PGD Hydrogels Crosslinked by Ethylene Glycol Diglycidyl Ether (EGDGE)

PGD-G3 and G4 hydrogels using EGDGE as a crosslinking agent (G3-EG and G4-EG hydrogels) were prepared by the following methods.

#### 4.2.1. PGD-G3 Hydrogels Prepared in NaOH Aqueous Solution [G3-EG(NaOH)]

PGD-G3 hydrogels were prepared by crosslinking using EGDGE in 1 M NaOH aqueous solutions at 60 °C overnight in a sample bottle (molding size: 14 × 3.5 mm). The detailed conditions and swelling properties are summarized in Table 1 and Figure 3. The number following (NaOH) in the sample code refers to the ratio of EGDGE and hydroxyl groups in one PGD-G3 molecule.

The swelling ratio was calculated by the following equation:

Swelling ratio (q) = W_s_/W_d_

where W_s_ is the weight of swollen hydrogel and W_d_ is the weight of the dried hydrogel.

#### 4.2.2. PGD-G3 Hydrogels Prepared in DMSO [G3-EG(DMSO)]

PGD-G3 hydrogels were prepared by crosslinking using EGDGE in DMSO in the presence of dimethylaminopyridine (DMAP) as a catalyst at 60 °C overnight. The detailed conditions and swelling properties are summarized in Table 1. The number following (DMSO) in the sample code refers to the ratio of EGDGE and hydroxyl groups in one PGD-G3 molecule. The swelling ratio was calculated by the method described above.

#### 4.2.3. PGD-G4 Hydrogels Prepared in NaOH Aqueous Solution [G4-EG(NaOH)]

In a similar manner to G3-EG hydrogels, PGD-G4 hydrogels using EGDGE as a crosslinking agent (G4-EG hydrogels) were prepared. The detailed conditions and swelling properties are summarized in Table 2.

### 4.3. PTX Loading of G3-EG and G4 Hydrogels

Each dried hydrogel was weighed to obtain the initial weight of the gel. PTX was dissolved in EtOH (1 mg/mL), and the hydrogels were placed in an EtOH solution of PTX for 2 days to allow the gels to reach swelling equilibrium. After two days, the gels were withdrawn from the PTX solution and stored in an oven (37 °C) until dry. After drying, acetonitrile was added to the gel to extract trapped PTX. The extraction % of PTX was more than 98% (9.0 μg/mL), because acetonitrile is a good solvent for PTX [45]. The PTX concentration was then measured using high-performance liquid chromatography (HPLC) (Agilent 1100 series) using a Symmetry column (Waters, Milford, MA, USA) at 25 °C. The mobile phase consisted of acetonitrile–water (45:55, v/v) with a flow rate of 1.0 mL/min. A diode array detector was used with a detection wavelength of 227 nm. The PTX concentrations in the samples were obtained from a calibration curve.

### 4.4. FTIR Imaging

The dried G4-EG(NaOH)1.0 hydrogel was sliced into samples with a thickness of about 0.1 mm. The sliced sample was placed on the sample holder of a multichannel infrared microscope system (FTIR-6200 with IMV-4000, JASCO, Tokyo, Japan). FTIR imaging was performed under the following conditions:

Objective focusing mirror magnification: ×16

Number of measurement points: 48 × 48

Measurement region: 1 point 12.5 × 12.5 µm (48 × 12.5 = 600 × 600 µm)

Number of integrations: 16

Resolution: 8 cm^−1^

### 4.5. PTX Release from G4-EG Hydrogels

The PTX-loaded G4-EG(NaOH)1.0 hydrogel was immersed in an aqueous solution. *N,N*-Diethylnicotinamide (1.5 M in PBS, pH 7.4) solution was used as the release medium to maintain an infinite sink without requiring simulated flow conditions [46]. Samples were taken at predetermined time intervals and assayed for PTX by isocratic reverse-phase HPLC (see Section 4.3).

## Figures and Tables

**Table 1:** Preparation conditions and swelling properties of PGD-G3 hydrogels.

Sample Code	Conc. of EGDGE (mmol/mL)	Conc. of PGD-G3 (wt. %)	EGDGE/OH Groups	Swelling Ratio (q)	
				In Water	In Ethanol
G3-EG(NaOH)0.15	0.682	32	0.15	5.39	1.16
G3-EG(NaOH)0.5	1.261	18	0.5	9.12	--
G3-EG(NaOH)0.75	1.215	11	0.75	36.20	4.28
G3-EG(DMSO)0.75	3.035	30	0.75	1.94	--
G3-EG(NaOH)1.0	1.621	11	1.0	11.39	3.83

**Table 2:** Preparation conditions and swelling properties of PGD-G4 hydrogels.

Sample Code	Conc. of EGDGE (mmol/mL)	Conc. of PGD-G4 (wt. %)	EGDGE/OH Groups	Swelling Ratio (q)	
				In Water	In Ethanol
G4-EG(NaOH)0.15	0.658	32	0.15	4.68	1.36
G4-EG(NaOH)0.5	1.217	18	0.5	7.41	2.05
G4-EG(NaOH)0.75	1.171	11	0.75	37.44	2.45
G4-EG(NaOH)1.0	1.564	11	1.0	9.95	2.14

## Data Availability

The dataset generated during the current study are not publicly available but are available from the corresponding author on reasonable request.
